# Monoaminergic Neuromodulation of Sensory Processing

**DOI:** 10.3389/fncir.2018.00051

**Published:** 2018-07-10

**Authors:** Simon N. Jacob, Hendrikje Nienborg

**Affiliations:** ^1^Department of Neurosurgery, Klinikum Rechts der Isar, Technical University of Munich, Munich, Germany; ^2^Werner Reichardt Centre for Integrative Neuroscience, University of Tübingen, Tübingen, Germany

**Keywords:** serotonin, dopamine, noradrenaline, primary sensory cortex, primary visual cortex (V1), primary auditory cortex, early sensory processing

## Abstract

All neuronal circuits are subject to neuromodulation. Modulatory effects on neuronal processing and resulting behavioral changes are most commonly reported for higher order cognitive brain functions. Comparatively little is known about how neuromodulators shape processing in sensory brain areas that provide the signals for downstream regions to operate on. In this article, we review the current knowledge about how the monoamine neuromodulators serotonin, dopamine and noradrenaline influence the representation of sensory stimuli in the mammalian sensory system. We review the functional organization of the monoaminergic brainstem neuromodulatory systems in relation to their role for sensory processing and summarize recent neurophysiological evidence showing that monoamines have diverse effects on early sensory processing, including changes in gain and in the precision of neuronal responses to sensory inputs. We also highlight the substantial evidence for complementarity between these neuromodulatory systems with different patterns of innervation across brain areas and cortical layers as well as distinct neuromodulatory actions. Studying the effects of neuromodulators at various target sites is a crucial step in the development of a mechanistic understanding of neuronal information processing in the healthy brain and in the generation and maintenance of mental diseases.

## Introduction

**FIGURE 1:**
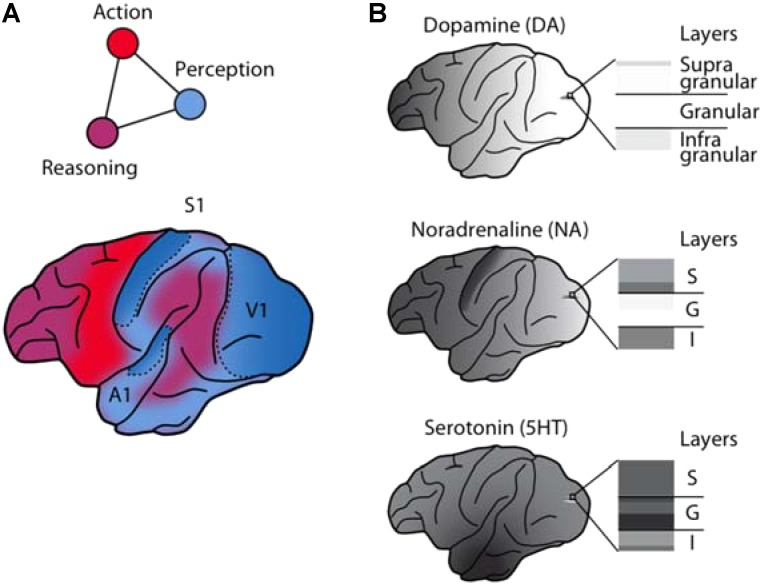
Anatomical and functional complementarity of the monoaminergic neuromodulators. **(A)** Schematic illustrating the functional divisions of the perception-action-reasoning cycle projected onto the lateral surface of a macaque brain [after (Fuster, 2015)]. **(B)** The monoaminergic neuromodulators show pronounced differences in cortical regional [after Brown et al. (1979)] and laminar innervation patterns.

Even at the earliest stages of sensory processing, the neuronal representation of external stimuli is modulated by internal brain states (Aston-Jones and Cohen, 2005; Harris and Thiele, 2011). While the evidence for such modulation is long-standing, the analysis of sensory representations has typically focused on the feed-forward stimulus-driven component while regarding the modulation by internal states as noise. However, recent results based on population recordings (Reimer et al., 2014; Rabinowitz et al., 2015; Schölvinck et al., 2015) that highlight the extent of brain-state dependent modulation of sensory processing, the discovery of substantial modulation of sensory activity with locomotion (Niell and Stryker, 2010; Polack et al., 2013), as well as novel tools to more selectively target modulatory circuit elements genetically have contributed to reviving the interest in the neuromodulation of sensory processing. Since the role of acetylcholine has received substantial attention and has been the subject of excellent recent reviews (Sarter et al., 2009; Harris and Thiele, 2011), we will focus here on the modulation by the monoamines dopamine (DA), noradrenaline (NA), and serotonin (5HT). For each of these modulatory systems, we will summarize anatomical data and electrophysiological findings to provide insights into their role in the modulation of sensory processing, placing an emphasis on studies in non-human primates and rodents, and highlighting the complementarity of these neuromodulatory systems (**Figure 1**). We will cover key classical studies and then shift our focus toward more recent work.

## Serotonergic Modulation of Sensory Processing

### Serotonin Sources

Serotonin-synthesizing neurons are located in the brainstem in a small group of clusters [named B1–B9 after Dahlström and Fuxe (1964)]. These were first identified in the rat brain (Dahlström and Fuxe, 1964) but their anatomical localizations in mouse and primate species, e.g., Jacobowitz and MacLean (1978), have been found to be largely consistent with that in the rat. These clusters are typically divided into a caudal and a rostral group (Hornung, 2010). The caudal group, which consists of the raphe pallidus (B1), the raphe obscurus (B2), and raphe magnus (B3) (Lesch and Waider, 2012), projects mainly to the spinal cord and brain stem. The rostral group includes the dorsal raphe nucleus (B6, B7) and the median raphe nucleus (B5, B8, and B9) and projects to the cortex. Within this group, there is some topographic organization of the serotonergic innervation (Hornung, 2010) that reflects a rostral (frontal cortex) to caudal (occipital cortex) gradient (Wilson and Molliver, 1991b). Such topographical organization suggests that rather than reflecting a signal that is broadcasted uniformly throughout the brain, serotonergic projections to different areas are more specific and may serve different roles. Indeed, retrograde studies in rats support topographically specific projections, for example to the prefrontal cortex (PFC) (Chandler et al., 2013). Although serotonergic neurons represent only a very small number of the neurons in the brain [approx. 28,000 in the mouse (Ishimura et al., 1988) to several 100,000 in humans (Hornung, 2010)], they give rise to diverging projections to virtually all regions of the mammalian brain (Hornung, 2010). Here, we will focus on the serotonergic projections to primary sensory cortical areas and subcortical structures involved in sensory processing.

### Serotonergic Projections to Early Sensory Areas

Anatomical studies in different rodent and monkey species have identified substantial serotonergic projections from the raphe nuclei to early sensory areas including the primary auditory, visual, and somatosensory areas, the olfactory bulb, and subcortical structures involved in sensory processing (**Table 1**). [Note that while in the auditory system, serotonergic innervation of the cochlear nucleus is established (Klepper and Herbert, 1991), serotonergic projections to the retina have been controversial (Schnyder and Künzle, 1984; Frazão et al., 2008)].

**Table 1:** Anatomical findings for the serotonin system in early sensory areas.

Sensory area	Species	Significance	Reference
V1	Squirrel monkey	V1 receives substantial projections from medial and dorsal raphe nucleus. Serotonergic fibers preferentially target layer 4 in V1.	Morrison et al., 1982a; Tigges et al., 1982
V1	Macaca fascicularis and nemestria	V1 receives substantial projections from the dorsal and medial raphe nuclei.	Doty, 1983
V1	Macaca fascicularis	Serotonergic fibers most pronounced in the layers 3–4Cα in V1 and overall denser than NA fibers	Kosofsky et al., 1984; de Lima et al., 1988
V1	Macaca fuscata	Serotonergic varicosities are densest in layer 4Cα, and in contact with stellate and pyramidal neurons.	Takeuchi and Sano, 1984
V1	Macaca fuscata and fascicularis	Detailed laminar profile of 5HT receptor expression. 5HT1_B_ and 5HT2_A_ are most pronounced. Expressed in layers 2–6, most pronounced in layer 4A and 4Cα/β.	Watakabe et al., 2009
V1	Rat	Transient increase during development in layer 4; later fairly uniform serotonergic innervation across layers.	Dori et al., 1996
V1	Rat	Serotonergic fibers in all layers but most pronounced in layer 4. Target pyramidal and interneurons. Among inhibitory interneurons, mostly somatostatin and NPY+ neurons but VIP+ interneurons are avoided.	Paspalas and Papadopoulos, 2001
LGN	Rat	Serotonergic projections identified using retrograde labeling; dense immunolabelling for the serotonin transporter (SERT), a sensitive marker for serotonergic fibers; most pronounced in the vLGN and IGL of the LGN complex	Villar et al., 1988; Papadopoulos and Parnavelas, 1990; Vertes et al., 2010
S1	Mouse	5HT3_A_ receptor is expressed on most non-PV, non-SST inhibitory interneurons	Rudy et al., 2011
S1	Mouse	Innervation across all layers in the adult. Transient increase during postnatal development (∼PD7). Similar to the rat.	Fujimiya et al., 1986
S1	Macaca fascicularis	(Areas 1, 2, 3) 5HT fibers across all layers but least pronounced in the lower part of layer 3 and layer 4.	DeFelipe and Jones, 1988
S1	Macaca mulatta and fascicularis	Fairly uniform distribution of serotonergic fibers across layers in S1.	Wilson and Molliver, 1991a,b
S1	Rat	5HT fibers are most pronounced superficially, but some controversy; 5HT concentration measured voltammetrically is highest in superficial layers and decreases toward deeper layers; Transient increase in layer 4 during development, later fairly uniform.	Fuxe, 1965; Beaudet and Descarries, 1976; Lamour et al., 1983; Dori et al., 1996
Inferior colliculus		Dense but complementary 5HT and NA projections in both IC and cochlear nucleus.	Klepper and Herbert, 1991
A1	Cat	5HT innervation mostly restricted to layers 1–3.	DeFelipe et al., 1991
A1	Macaca fascicularis	“Uniformly high density” across all layers.	Campbell et al., 1987
MGN	Rat	Homogenous serotonergic innervation.	Vertes et al., 2010
SC	Rat	Serotonergic fibers throughout, but more pronounced in the superficial than in the deep layers of the SC.	Villar et al., 1988; Dori et al., 1998
Olfactory bulb	Rat	Projections from dorsal and median raphe to all layers of olfactory bulb; most densely in glomerular layer, i.e., the input layer.	McLean and Shipley, 1987


Comparisons of monoaminergic innervation found that the serotonergic innervation was substantially more pronounced than that for NA or DA in the macaque primary auditory cortex (Campbell et al., 1987) and in the macaque primary visual cortex (Morrison et al., 1982a; Kosofsky et al., 1984; Morrison and Foote, 1986; Lewis et al., 1987). This pattern mirrors early reports of regional differences in cortical monoaminergic distribution (Brown et al., 1979), with a pronounced decrease in DA from frontal to occipital cortex, a weaker gradient (interrupted by a peak in the somatosensory cortex) for NA, and a roughly uniform distribution for serotonin (**Figure 1B**).

Within primary sensory cortical areas, the distribution of serotonergic fibers in primates shows a characteristic laminar profile. In the primary visual cortex, anatomical data for different primate species agree that the distribution of serotonergic axons is highest in layer 4 (Kosofsky et al., 1984; Morrison and Foote, 1986). This suggests that the serotonergic modulation in the primary visual cortex is biased toward targeting the visual input stage. Serotonergic fibers are also consistently found in cortical layer 4 in the primary auditory cortex (Campbell et al., 1987) and somatosensory cortex (Wilson and Molliver, 1991a,b), although not preferentially.

This contrasts with the monoaminergic innervation of primary sensory areas by NA and DA (see below) that is typically sparse if not absent in layer 4 (**Figure 1B**). (Note that while a transient dominance of serotonergic input to layer 4 occurs during the early post-natal development in the rodent primary visual cortex and barrel cortex (Fujimiya et al., 1986; Dori et al., 1996), regional and laminar differences are less pronounced in rodents than in the primate species.)

Consistent serotonergic innervation is also found in subcortical structures involved in sensory processing such as the sensory thalamus (Vertes et al., 2010), the superior (Dori et al., 1998) and inferior (Klepper and Herbert, 1991) colliculi, and the cochlear nucleus (Klepper and Herbert, 1991). In the olfactory bulb, serotonergic fibers reach the glomerular layer, the primary input stage (Takeuchi et al., 1982). Together, the extent of serotonergic innervation at the earliest stages of sensory processing makes this system well suited to directly modulate the incoming sensory information.

### Serotonergic Synapses and Receptors

The serotonergic projections to the primary sensory areas consist of small varicose axons that are widely distributed (Hornung, 2010). Only a very small proportion of synaptic specializations, typically asymmetric, is found (Descarries et al., 2010), suggesting that serotonin predominantly acts by volume transmission from varicosities. Note, however, that the degree to which neuromodulatory systems rely on “wired” transmission, i.e., highly localized and typically synaptic, or “volume” transmission, i.e., more spatially diffuse, is subject to debate (e.g., Rice and Cragg, 2008; Sarter et al., 2009; Fuxe and Borroto-Escuela, 2016; Sulzer et al., 2016). In the mammalian brain, seven serotonin receptor families, most with several subtypes, have been identified to date and contribute to the functional diversity of serotonin (Mengod et al., 2010). A detailed overview is outside the scope of this review, but a few receptors should be highlighted. 5HT1A is expressed on cortical pyramidal neurons (DeFelipe et al., 2001). In the macaque primary visual cortex, the most densely expressed receptors are 5HT1B and 5HT2A (Watakabe et al., 2009), predominantly in layer 4. 5HT1B is also strongly expressed in the LGN, but only weakly in other cortical areas including the auditory and somatosensory cortex (Watakabe et al., 2009). In the mouse, GABAergic neurons that express the 5HT3A do not express the calcium binding protein parvalbumin or somatostatin and may form a third non-overlapping class of inhibitory interneurons (Rudy et al., 2011).

### Serotonergic Modulation of Sensory Physiology

Given the complexity of the input to serotonergic neurons (Pollak Dorocic et al., 2014), the wide distribution of serotonergic projections in the brain and the diversity of serotonin receptors, it is unsurprising that serotonin has been implicated in a spectrum of brain functions. These include the sleep–wake cycle, hormonal regulation, regulation during development as well as affective, cognitive, and sensorimotor functions. Serotonergic neurons show tonic and phasic modes of discharge that are thought to signal different information. For example, in the mouse transient responses of putative serotonergic neurons in the dorsal raphe nucleus reflect a variety of behavioral, sensorimotor, and reward-linked information (Ranade and Mainen, 2009). Phasic and tonic response patterns of optogenetically identified serotonergic neurons in the mouse dorsal raphe have been proposed to reflect the contextual valence on different time-scales (Cohen et al., 2015). Serotonergic neurons have also been linked to signaling patience (Fonseca et al., 2015). But, even for the intensely studied links to reward signaling a simple computational account has proved challenging (Ranade et al., 2014; Dayan and Huys, 2015). Although it is unclear whether its modulatory role can be conceptualized by a simple overarching computational function, serotonin is thought to modulate sensory processing according to behavioral–motivational context.

In reviewing studies of serotonergic modulation of early sensory processing across modalities, consistencies are notable, which will be our focus here (**Table 2**). This focus contrasts with previous perspectives highlighting the diversity of findings (Hurley et al., 2004). (Note that we restricted this summary to studies of short-term sensory modulation and did not consider reports on adaptation or plasticity.) Some variability in the findings likely results from comparing results across anesthetized animals using different anesthetics or awake animals and different experimental approaches.

**Table 2:** Findings related to sensory modulation by serotonin.

Sensory area	Species and anesthesia, if applicable	Significance	Reference
Cochlear nucleus	Urethane anesthetized rat	Mostly suppressive effect of iontophoretically applied 5HT on extracellular responses	Ebert and Ostwald, 1992
Inferior colliculus	Lightly anesthetized (ketamine/xylazine) Bat	Multiplicative decrease of response with iontophoretic 5HT application; weak increase in latency	Hurley and Pollak, 1999, 2005
Putative auditory contribution	Behaving rat	Startle response to auditory white noise is reduced by intraventricular 5HT application	Davis et al., 1980
V1	Anesthetized (isoflurane/droperidol/fentanyl) macaque	Variable bidirectional modulation of extracellular responses with application of 5HT1_B_ and 5HT2_A_ receptor selective agents	Watakabe et al., 2009
V1	Anesthetized (fentanyl/thiopental/succinylcholine chloride) macaque	Intracortical injection of 5HT1_A_ agonist decreases extracellularly recorded responses	Rauch et al., 2008
V1	Behaving macaque	Gain decrease of extracellular responses with iontophoretic 5HT application. Modest increase in response latency. No systematic effect on response variability, co-variability, or selectivity	Seillier et al., 2017
V1	Halothane anesthetized rat	Variable findings for iontophoretic 5HT application but mainly a decreased response across the population	Waterhouse et al., 1990
dLGN	Halothane/nitrous oxide anesthetized cat	Decrease of the extracellular responses for iontophoretic 5HT application	Phillis et al., 1967
S1	Halothane anesthetized rat	Suppression of response to tactile stimuli (forepaw touch), consistent with response gain decrease with iontophoretic 5HT application	Waterhouse et al., 1986
	Behaving mouse	Decreased mechanosensory response of mice during optogenetic activation of serotonergic raphe neurons	Dugué et al., 2014
	Behaving macaque	Blocking serotonin re-uptake slowed reaction times and worsened perceptual performance in a visual (color) discrimination task	Costa et al., 2016
Olfactory bulb	Ketamine/xylazine anesthetized mouse	Gain decrease for application of 5HT agonists, reversed for application of 5HT antagonist.	Petzold et al., 2009
Olfactory bulb	Urethane anesthetized mouse	Optogenetic activation of serotonergic neurons in the dorsal raphe lead to a gain decrease of spontaneous but not stimulus-driven extracellular response in the OB resulting in an increase in SNR	Lottem et al., 2016


Despite this variability, most findings are consistent with an overall serotonin-mediated decrease of the sensory response. In the auditory system, a decrease of the extracellular response was observed for the majority of neurons both in the cochlear nucleus of the rat (Ebert and Ostwald, 1992) and the inferior colliculus (IC) of bats (Hurley and Pollak, 1999), although some variability of the effects between cells was observed. Behaviorally, a reduced startle response to auditory tones was observed for intraventricular injection of serotonin (Davis et al., 1980), which is consistent with reduced auditory response (but could also reflect downstream processing). Similarly, a reduced mechanosensory response was observed in behaving mice during optogenetic stimulation of serotonergic raphe neurons (Dugué et al., 2014), which could reflect downstream processing but would also be expected for a reduced sensory response in the somatosensory cortex as previously reported (Waterhouse et al., 1986).

For early visual processing, predominantly decreased responses were observed for the iontophoretic application of serotonin in the cat lateral geniculate nucleus (LGN) (Phillis et al., 1967) and rat V1 (Waterhouse et al., 1990). In the anesthetized macaque, when attempting to dissect the role of the two most strongly expressed receptors in V1, 5HT1B and 5HT2A, using receptor specific ligands, a diverse pattern and bi-directional modulation were observed for both (Watakabe et al., 2009). However, these effects were not compared to spontaneous variability of the responses resulting from the anesthesia or the iontophoretic application itself. For example, slow fluctuations in the neuronal responses have been shown to contribute to stimulus-independent co-variability (“noise correlations”) between neurons (Ecker et al., 2014). When instead iontophoretically applying the endogenous ligand serotonin in awake macaques and comparing the effects against those of pH matched saline application, a recent study found an overall decrease of the sensory responses with serotonin (Seillier et al., 2017). While there was some variability across cells, consistent with the results of Watakabe et al. (2009), the inhibitory effect of serotonin was the dominant pattern across the sizeable neuronal population. This decrease was predominantly explained by a multiplicative change (gain change) of the neuronal tuning curves. Behaviorally, a recent study that systemically administered a serotonin-reuptake inhibitor to enhance the effect of serotonin while macaques performed a color discrimination task observed slowed reaction times as well as deteriorated perceptual performance (Costa et al., 2016), as expected for such reduced visual responses. Conversely, a gain reduction of the spontaneous response (Lottem et al., 2016), consistent with an increased signal-to-noise ratio (SNR), or a gain reduction of the tuning curves (Petzold et al., 2009) for serotonin was observed in the mouse olfactory bulb.

Taken together, a surprisingly consistent pattern of the serotonergic modulation of early sensory processing across modalities emerges. The decreased sensory response by serotonin – effectively lowering the salience of the sensory input – may reflect a sensory signature of how serotonin shapes behavior in downstream circuits, such as its proposed role as a behavioral inhibitor (Soubrie, 1986), to promote waiting (Miyazaki et al., 2014; Ranade et al., 2014; Fonseca et al., 2015) or persistence (Lottem et al., 2018).

## Noradrenergic Modulation of Sensory Processing

### Noradrenergic Sources

In rodents (Dahlström and Fuxe, 1964) and primates (Jacobowitz and MacLean, 1978), neurons that produce NA are located in the brainstem in clusters named A1–A7 after (Dahlström and Fuxe, 1964). Of these, the locus coeruleus (LC, A6) projects to most areas in the brain (Loughlin et al., 1986), except for to the basal ganglia [reviewed in Berridge and Waterhouse (2003)], and is the sole source of the noradrenergic innervation of the cerebral cortex (Jones et al., 1977; Moore and Bloom, 1979). Immunohistochemical evidence indicates that within the LC the vast majority of neurons are noradrenergic (Grzanna and Molliver, 1980). Similar to the serotonergic and dopaminergic systems, the absolute number of noradrenergic neurons in the LC is small [estimated between approx. 1500 per hemisphere in rodents and approx. 15,000 per hemisphere in humans, reviewed in Sara and Bouret (2012)], but the projections of these neurons are divergent and wide-spread throughout the brain. Despite these wide-spread projections recent findings indicate a modular organization of the LC in rats (Uematsu et al., 2017) and substantial anatomical specificity of the connections, for example to the prefrontal versus the motor cortex (Chandler et al., 2014), or different modules projecting to the amygdala compared to the medial PFC (mPFC) (Uematsu et al., 2017). It therefore seems likely that at least some degree of modularity is also characteristic of projections to sensory areas. In the following, we will again focus on the projections to the primary sensory areas and subcortical structures involved in early sensory processing.

### Noradrenergic Projections to Early Sensory Areas

The LC sends divergent projections to the cortex and subcortical structures. Within the LC, these projections are roughly topographically organized [reviewed in Berridge and Waterhouse (2003)]. In the rat, cortex-projecting neurons are more prominent in the caudal portion of the LC and show some ventral (frontal cortex) to dorsal (occipital cortex) organization (Waterhouse et al., 1983). Despite the rough topography, a recent study combining retrograde labeling and optogenetic stimulation in the mouse found that the LC projections to primary sensory cortices are not modality-specific (Kim et al., 2016) (contrasting with these authors’ findings for the cholinergic system). Nonetheless, projections from LC show clear regional differences (**Table 3**). Early studies in rats (Kehr et al., 1976) and macaques (Brown et al., 1979) observed an overall frontal (higher concentration) to occipital (lower concentration) gradient of NA throughout cortex, with the exception of somatosensory cortex where the concentration was highest (Brown et al., 1979). This gradient was weaker than that for DA and differed markedly from the absence of a gradient for serotonin (reviewed above and see **Figure 1**).

**Table 3:** Anatomical findings for the NA system in early sensory areas.

Structure	Species	Significance	Reference
V1	Squirrel monkey	Noradrenergic projections preferentially to layers 5 and 6 of V1	Morrison et al., 1982a; Lewis et al., 1987
V1	Macaca fascicularis	NA innervation much sparser than for 5HT, and least dense in layer 4C	Kosofsky et al., 1984
V1	Cynomolgus monkey	NA fibers less pronounced than 5HT. Weak in layers 1 and 2, and absent in layer 4Cβ	Kosofsky et al., 1984
V1	Rat	Decreasing fronto-occipital gradient of NA	Kehr et al., 1976
LGN	Squirrel monkeys and macaca fascicularis	Almost no noradrenergic innervation in the LGN	Morrison and Foote, 1986
LGN	Rat	NA fibers preferentially in the dLGN compared to vLGN and IGL (compared to 5HT fibers preferentially in the vLGN and IGL)	Papadopoulos and Parnavelas, 1990
S1	Squirrel monkey	Labeling in all layers	Morrison et al., 1982b; Lewis et al., 1987
S1	Rhesus monkeys	NA concentration highest in somatosensory cortex, lowest in V1	Brown et al., 1979
A1	Macaca fascicularis	Sparse innervation (substantially less dense than 5HT or Ach), lowest density in layer 4	Campbell et al., 1987
Olfactory bulb	Rat	Virtually no labeling fibers in the glomerular layer (first input stage) but fibers preferentially in the internal plexiform, granule cell, and external plexiform layers	McLean et al., 1989
IC, CN	Rat	LC heavily innervates both structures	Klepper and Herbert, 1991


Within the primary sensory cortex, the noradrenergic innervation shows some laminar specialization, particularly in primates. In the primate somatosensory cortex, noradrenergic fibers are fairly uniform and dense throughout layers (Morrison et al., 1982b; Lewis et al., 1987). The innervation of the primary auditory cortex (Campbell et al., 1987) and primary visual cortex (Kosofsky et al., 1984; Morrison and Foote, 1986), in contrast, is sparser overall, and a “striking absence” (Foote and Morrison, 1987) of layer 4 innervation, particularly in V1, has been observed. Combined with the near absent innervation of the LGN (Morrison and Foote, 1986), which provides the dominant feed-forward visual input to V1, this suggests that – in contrast to the serotonergic system – noradrenergic modulation does not target the visual input stage. Interestingly, such complementarity between the serotonergic and noradrenergic innervation of the sensory input stage is also present in the rat olfactory bulb. While “virtually no label” was observed in the glomerular layer (the site of the first synapse of the olfactory input) using anterograde tracer injections in the LC (McLean et al., 1989), this layer showed the densest serotonergic innervation (McLean and Shipley, 1987) (see above). Instead, pronounced noradrenergic innervation in the rat olfactory bulb was observed for the consecutive processing stages, the internal plexiform, granule, and external plexiform layers (McLean et al., 1989).

### Noradrenergic Synapses and Receptors

Similar to the other monoaminergic systems, noradrenergic neurons in the LC show both tonic and phasic modes of activation [reviewed in Berridge and Waterhouse (2003)]. Their axons have characteristic NA-containing varicosities that can form synapses (e.g., Papadopoulos et al., 1989) and likely have non-synaptic release sites (Callado and Stamford, 2000). Three adrenergic receptor families are expressed in the brain (alpha 1, alpha 2, and beta 1–3), which have characteristic pre- and postsynaptic sites and laminar expression patterns across cortex (Berridge and Waterhouse, 2003). Noradrenaline has the highest affinity for the α2 receptors, intermediate for α1 receptors, and lowest affinity for the β adrenergic receptors [reviewed in Ramos and Arnsten (2007)]. In the primate PFC, these differences in affinity have been implicated in differentially modulating cognitive processes as a function of the level of noradrenergic tone (Ramos and Arnsten, 2007). They are likely also an important factor in the substantial diversity in modulation of sensory processing observed for NA (see below).

### Noradrenergic Modulation of Sensory Physiology

The brain’s noradrenergic system has long been linked to arousal (e.g., O’Hanlon, 1965; reviewed in, e.g., Berridge and Waterhouse, 2003; Sara and Bouret, 2012) and to adapting network activity for optimal, flexible behavior (Aston-Jones and Cohen, 2005; Bouret and Sara, 2005). Consistent with such a general function, NA has been shown to modulate sensory processing in complex ways (**Table 4**).

**Table 4:** Findings related to sensory modulation by NA.

Sensory area	Species and anesthesia (if applicable)	Significance	Reference
dLGN	Chloral hydrate/urethane anesthetized rat	NA or LC activation facilitates responses in dLGN; the facilitation of LGN response to LC stimulation is blocked by α1 but not β receptor antagonists	Rogawski and Aghajanian, 1980; Kayama et al., 1982
dLGN, dorsal, and ventral thalamus	Halothane anesthetized cat	Predominantly depression of responses by iontophoretic application of NA	Phillis et al., 1967; Phillis and Teběcis, 1967
V1	Nitrous oxide anesthetized cat	Bi-directional modulation. Enhanced SNR for modulated neurons	Kasamatsu and Heggelund, 1982
V1	Halothane anesthetized/nitrous oxide cat	LC stimulation results in both facilitation and inhibition of extracellular responses in V1, which vary by layer. Results from α1, α2, and β antagonists suggest that α receptors facilitate the responses while β receptor activation results in bi-directional modulation. No change in SNR	Sato et al., 1989
V1	Thiopental anesthetized cat	Iontophoretic NA application results in variable modulation of the responses and affects receptive field properties	McLean and Waterhouse, 1994
V1	Awake mouse	NA was found necessary for tonic depolarization with locomotion of layer 2/3 excitatory neurons	Polack et al., 2013
V1	Urethane anesthetized rat	LC activity precedes increases in cortical excitability	Safaai et al., 2015
V1	Halothane anesthetized rat	Iontophoretic application of NA enhances visual responses	Waterhouse et al., 1990
A1	Awake squirrel monkey	Iontophoretic NA application decreases evoked and spontaneous extracellular activity consistent with an increased SNR	Foote et al., 1975
A1	Urethane-anesthetized rat	Iontophoretic NA application induces bi-directional modulation, with the dominant effect a response decrease, α1-receptor mediated. No net change in SNR across the population	Manunta and Edeline, 1997
CN	Awake bat	Enhances “temporal contrast,” i.e., the temporal precision of the response	Kössl and Vater, 1989
S1 and ventral posteriomedial thalamus	Awake rat	Tonic vs. phasic activation differentially modulates responses in the somatosensory processing hierarchy	Devilbiss and Waterhouse, 2011
S1	Isoflurane anesthetized rat	Intracellular recordings *in vivo*: NA blockage reduces up-states in neurons	Constantinople and Bruno, 2011
S1	Urethane anesthetized and awake rat	Iontophoretic NA application suppresses spontaneous and glutamate evoked activity	Armstrong-James and Fox, 1983; Bassant et al., 1990
S1	Halothane anesthetized rat	Variable effects on rates and SNR for iontophoretic NA application; phasic LC stimulation predominantly enhances responses. NA depletion abolishes this modulation	Waterhouse and Woodward, 1980; Waterhouse et al., 1980, 1998
Piriform cortex	Urethane anesthetized rat	Mainly enhancement of odor responses in piriform cortex with LC stimulation	Bouret and Sara, 2002
Olfactory bulb	Urethane anesthetized rat	Infusion of NA and stimulation of the LC decreases responses at low and high levels but not intermediate levels of stimulation/NA	Manella et al., 2017


Earlier findings have been reviewed in detail (Berridge and Waterhouse, 2003). In brief, activating the LC or local application of NA has been found to result in a variety of effects, including inhibitory and/or facilitating modulation, selective gating, changes to a neuron’s receptive field, and changes to its SNR. For example, iontophoretic application of NA was found to predominantly decrease responses in the dLGN, dorsal, and ventral thalamus of cats (Phillis et al., 1967; Phillis and Teběcis, 1967), in A1 of squirrel monkeys (Foote et al., 1975) and of rats (Manunta and Edeline, 1997), and in rat S1 (Armstrong-James and Fox, 1983; Bassant et al., 1990). Conversely, a predominantly facilitating effect was observed for iontophoretic application of NA or LC stimulation in the rat dLGN (Rogawski and Aghajanian, 1980; Kayama et al., 1982) or V1 (Waterhouse et al., 1990), and for phasic LC stimulation in rat S1 (Waterhouse and Woodward, 1980; Waterhouse et al., 1980, 1998) or rat piriform cortex (Bouret and Sara, 2002). In cat V1 Sato and colleagues (Sato et al., 1989), observed a substantial layer-dependence of the response modulation with LC stimulation. It was predominantly inhibitory in layers 2–4, mostly α receptor mediated facilitation in layer 5 and approximately balanced in both directions in layer 6. These laminar differences likely reflect different receptor expression profiles across layers. The results suggest that some of the variability between studies may also reflect laminar differences between studies within the targeted structures in addition to, e.g., dose-dependent effects. Mirroring this variability, noradrenergic effects on the neuronal SNR differed between studies. While some reported an enhanced SNR (e.g., Foote et al., 1975; Waterhouse and Woodward, 1980; Kasamatsu and Heggelund, 1982) and others (Sato et al., 1989; Manunta and Edeline, 1997) reported no net effect on SNR across the population. In line with the notion of optimizing the neuronal gain for behavior (Aston-Jones and Cohen, 2005), this variability may reflect the noradrenergic role in different states of a dynamical system.

Indeed, simultaneous recordings in the LC and barrel cortex of anesthetized rats combined with dynamical systems modeling showed that activity in the LC was highly predictive of dynamic changes in cortical excitability (Safaai et al., 2015). Moreover, phasic responses in the LC or urethane anesthetized rats were elicited by aversive somatosensory stimuli and modulated the stimulus-induced gamma oscillations in the mPFC, suggesting a corresponding modulation of somatosensory processing (Neves et al., 2018). In awake mice, visual responses have been found to be substantially enhanced during phases when the animals were running compared to no locomotion (Niell and Stryker, 2010). Intriguingly, intracellular recordings showed that this effect was accompanied by a depolarization of the membrane potential in layer 2/3 neurons, which in turn was blocked by noradrenergic antagonists (Polack et al., 2013). Indeed, both noradrenergic and cholinergic mechanisms were linked to this locomotion-dependent modulation, with partially complementary functions (Polack et al., 2013; Reimer et al., 2016). These findings align with the notion of a noradrenergic role in adapting sensory circuits for optimal behavior (Aston-Jones and Cohen, 2005). Additionally, they highlight the importance of interactions between neuromodulatory systems that go beyond the scope of this review.

## Dopaminergic Modulation of Sensory Processing

### Dopamine Sources

Dopamine synthesizing neurons represent a comparatively small population of neurons in the mammalian brain (Bentivoglio and Morelli, 2005). The majority of DA neurons are found in three cell groups in the ventral midbrain (mesencephalon) (Dahlström and Fuxe, 1964). Based on cytoarchitectonic features, most dopaminergic cells reside in the substantia nigra (SN) pars compacta (SNc, A9), the ventral tegmental area (VTA, A10) medial to the SN and in the retrorubral area (RRA; A8), which lies caudal and dorsal to the SN. Other, smaller dopaminergic cell groups are present in the periaqueductal gray matter (PAG; A11) and in the hypothalamus (A12–A15), the lateral parabrachial nucleus, the olfactory bulb (A16), and in the retina (A17) (Sánchez-González et al., 2005).

The most frequently used marker for identifying dopaminergic neurons is tyrosine hydroxylase (TH), the rate-limiting enzyme of DA synthesis. For TH-immunopositive neurons, the A8 group comprises approx. 5% of cells, while A9 and A10 account for the remaining 95% with a slight dominance of A9 over A10 (Bentivoglio and Morelli, 2005). The DA system undergoes considerable expansion from rodents to primates (Berger et al., 1991). By immunostaining for TH, the number of mesencephalic DA neurons has been estimated at approx. 20,000–30,000 in total in mice (Nelson et al., 1996), 45,000 in rats (German and Manaye, 1993), 110,000–220,000 in the SN in rhesus monkeys (Emborg et al., 1998), and between 230,000 and 430,000 in the human SN (Chu et al., 2002). Thus, a large dopaminergic A9 group is a distinctive feature of the primate brain.

Tyrosine hydroxylase labeling to identify dopaminergic neurons has been called into question because of a lack of specificity (Lammel et al., 2015). Staining for the dopamine transporter (DAT), which removes DA from the extracellular space and is largely responsible for the termination of dopaminergic neurotransmission, is more specific and is not detected, e.g., in noradrenergic cells (Ciliax et al., 1995). However, DAT is not expressed in all DA neurons. For example, it cannot be used to label the diencephalic (hypothalamic) cell groups (Sánchez-González et al., 2005). The subgroup of VTA DA neurons that project to the PFC also contains very little DAT (Lammel et al., 2008). Distinct expression profiles of TH and DAT can therefore be exploited to trace independent populations among dopaminergic neurons.

There is mounting evidence that DA is also released from terminals of LC neurons as a co-transmitter of NA (Devoto et al., 2001, 2003, 2005). These important findings underscore the potential caveats of using cellular protein markers instead of the transmitter receptor or the transmitter itself to investigate monoaminergic neurotransmission. Considerable complexity within the monoaminergic system is therefore likely to shape sensory and higher-order cognitive processing (Takeuchi et al., 2016).

### Dopamine Projections

A variety of different techniques has been used to investigate dopaminergic innervation of target structures, including direct (e.g., photometric or autoradiographic) neurotransmitter measurements (Brown et al., 1979; Descarries et al., 1987), immunostaining for TH in comparison to staining for DA-β hydroxylase (Lewis et al., 1987) and DAT (Sánchez-González et al., 2005), DA receptor autoradiography (Lidow et al., 1991), and DA receptor mRNA assays (Weiner et al., 1991; Hurd et al., 2001; Santana et al., 2009). It is important to keep in mind that these methods differ not just with regard to their sensitivity and specificity, but that they also target distinct stages of DA production and neurotransmission. This is a likely source of variability and even discrepancy in the literature. Ultimately, functional evidence is required that local DA modulates neuronal processing in target areas. Physiological studies are therefore the gold-standard for demonstrating effective dopaminergic innervation (see below).

The DA system projects extensively to many subcortical and cortical structures of the brain, albeit with a clear concentration. Lewis et al. (1987) noted, summarizing their work on the cortical distribution of dopaminergic afferents in the non-human primate, that “dopaminergic fibers preferentially innervate motor over sensory regions, sensory association over primary sensory regions, and auditory association over visual association regions.”

The dopaminergic projections from the midbrain are typically subdivided into the mesostriatal pathway and the mesocorticolimbic pathway. The striatum is the major target of mesencephalic DA neurons (Bentivoglio and Morelli, 2005). Ipsilateral projections to the dorsal striatum mainly arise from the SNc (nigrostriatal pathway). Limbic efferents including projections to the ventral striatum (nucleus accumbens) emanate largely from the VTA.

The mesocortical pathways target in particular the motor cortex, the prefrontal, the anterior cingulate, and the rhinal cortices in rodents (Descarries et al., 1987) and in the non-human primate (Berger et al., 1988; Lidow et al., 1991). Similar to the number of dopaminergic midbrain neurons, however, the density of cortical DA terminals varies considerably from species to species, reaching a maximum in primates (Berger et al., 1991). Unlike NA and, especially, serotonin, cortical levels of DA in the macaque brain exhibit a prominent gradient with a strong decrease along the fronto-occipital axis and only minimal amounts detectable in visual cortex (Brown et al., 1979; see **Figure 1B**).

Mesocortical projections originate from all major cell groups in the ventral mesencephalon, with a looser organization compared to the mesostriatal system. A clear topographical relationship, however, has been described for the most densely innervated frontal lobe. Dopaminergic projections to the dorsolateral PFC in the macaque brain arise from the dorsal and lateral regions of three midbrain cell groups A8, A9, and A10, whereas the ventromedial regions of the PFC receive projections from the medial parabrachial pigmented nucleus and the midline nuclei of the VTA (Williams and Goldman-Rakic, 1998). A similar medial–lateral arrangement has been found in rats (Loughlin and Fallon, 1984). Here, most of the dopaminergic innervation of the medial frontal cortex comes from VTA neurons that project to the deep layers 5 and 6 of the mPFC and in particular to the orbitofrontal cortex (Chandler et al., 2013), with substantially fewer cells from the SNc innervating predominately the superficial mPFC layers (Berger et al., 1991). SNc DA neurons preferentially target the lateral frontal cortex, e.g., the premotor areas (Loughlin and Fallon, 1984; Muller et al., 2014).

Overall, VTA dopaminergic neurons show little collateralization to extensive terminal fields, somewhat in contrast to SNc cells (Loughlin and Fallon, 1984; Chandler et al., 2013). This suggests that, in light of the topographical arrangement described above, discrete, anatomically circumscribed dopaminergic subsystems exist that could modulate selected target regions.

### Dopamine Synapses and Receptors

Dopamine-containing varicosities can form multiple synaptic contacts along the course of an axonal fiber. In frontal cortex, these terminals establish conventional, symmetric synapses with postsynaptic dendritic shafts and spines, preferentially on pyramidal neurons (Berger et al., 1991). At the ultrastructural level, dopaminergic afferents form synaptic triads with postsynaptic spines that receive a second, presumably glutamatergic input (Goldman-Rakic et al., 1989). This configuration would allow DA to modulate ongoing neuronal transmission both pre- and postsynaptically.

Dopamine receptors are expressed by pyramidal neurons and GABAergic interneurons alike, indicating that DA modulates excitatory and inhibitory synaptic transmission (Santana et al., 2009). Dopamine receptors are categorized into two major families: the D1 family comprising the D_1_ and D_5_ receptors, and the D2 family comprising the D_2_, D_3_, and D_4_ receptors. Both D1 and D2 families of receptors are G protein-coupled receptors, which initiate intracellular signaling cascades rather than directly inducing postsynaptic currents (Missale et al., 1998; Bentivoglio and Morelli, 2005). D1 and D2 receptors are largely expressed in different cell populations (Vincent et al., 1993). In most rat brain structures except for the VTA and some midbrain cell groups, D1 receptors outnumber D2 receptors (Boyson et al., 1986; Weiner et al., 1991). In the human brain, D1 receptor mRNA clearly dominates over D2 receptor mRNA in the cortical mantle, whereas D2 receptor mRNA is more abundant in the hippocampal formation, brainstem, and in subcortical structures such as the thalamus (Hurd et al., 2001). Of note, a high proportion of DA (D1) receptors are found at extrasynaptic sites, suggesting that DA might also exert its effects via diffusion in the neuropil (volume transmission) (Smiley et al., 1994).

Cortical DA receptors show a laminar-specific distribution profile. D1 receptors are expressed in all cortical layers in the primate frontal brain (Lidow et al., 1991; Huntley et al., 1992), often with a bilaminar infra-supragranular pattern with a relative paucity in layer 4 (Lewis et al., 1987) and a predilection for deeper layers. D2 receptors, in contrast, are typically confined to cortical layer 5 (Weiner et al., 1991; Lidow et al., 1998).

### Dopaminergic Innervation of Sensory Brain Structures

Compared to the densely innervated frontal cortex, the sensory cortices receive very sparse dopaminergic afferents (**Table 5**). In both rat and primate studies, the primary visual cortex shows the lowest DA fiber density of all investigated cortical structures (Descarries et al., 1987; Lidow et al., 1991). TH immunoreactivity in primate primary visual cortex V1 is restricted to layer 1, and few fibers reach layer 6 as well in V2 (Lewis et al., 1987; Berger et al., 1988). The same layers are targeted by dopaminergic fibers in the rat primary visual cortex (Phillipson et al., 1987).

**Table 5:** Anatomical findings for the DA system in early sensory areas.

Structure	Species	Significance	Reference
V1	Rhesus monkey and rat	Lowest density of dopaminergic innervation across cortical mantle	Brown et al., 1979; Descarries et al., 1987; Lidow et al., 1991
V1	Cynomolgus monkey	TH immunoreactive fibers restricted to layer 1	Berger et al., 1988
V1	Rat	Dopaminergic innervation in infragranular layers and weaker in layer 1	Phillipson et al., 1987
A1	Gerbil	D1 receptors in infragranular layers	Schicknick et al., 2008
A1	Cynomolgus and squirrel monkey	TH immunoreactive fibers in layers 1 and 6	Lewis et al., 1987
S1	Cynomolgus and squirrel monkey	TH immunoreactive fibers in supra- and infragranular layers	Lewis et al., 1987
LGN	Rat	Dopaminergic innervation of all LGN subdivisions	Papadopoulos and Parnavelas, 1990
LGN and MGN	Human	Moderate levels of D2 receptor mRNA	Hurd et al., 2001
Inferior colliculus	Rat and mouse	Innervation by TH immunoreactive fibers	Tong et al., 2005; Nevue et al., 2015
Inferior colliculus	Rat	D2 receptors present at moderate levels	Weiner et al., 1991
Superior colliculus	Rat	D2 receptors present at low levels	Weiner et al., 1991


A slight increase in fiber density is seen in primary auditory cortex. In the gerbil, D1 receptors are found in infragranular layers mainly associated with pyramidal neurons (Schicknick et al., 2008). TH positive fibers loosely innervate auditory cortex layers 1 and 6 in primates (Lewis et al., 1987). In primary somatosensory cortex, labeled fibers are also found in layer 5. A further increase in dopaminergic innervation is then observed in the association cortices of the temporal, parietal, and frontal lobe, where the aforementioned bilaminar profile emerges (Lewis et al., 1987).

Dopaminergic fibers have also been reported to innervate subcortical structures that are involved in processing sensory stimuli. Dopamine axons reach the LGN of the thalamus (LGN, visual nucleus) (Papadopoulos and Parnavelas, 1990). Here, D1 and D2 receptors are found in excitatory relay neurons and in inhibitory local interneurons (Albrecht et al., 1996; Zhao et al., 2002; Munsch et al., 2005). D2 receptors are also present in the thalamic medial geniculate nucleus (MGN, auditory nucleus) (Hurd et al., 2001; Chun et al., 2014) and in the ventrobasal complex (VB, somatosensory nucleus) (Govindaiah et al., 2010b).

While these first-order thalamic nuclei mainly relay sensory input to the cortex, the second-order thalamic nuclei are part of cortico–thalamo–cortical loops and could support higher brain functions operating on sensory information (Sherman, 2016). The second-order thalamus is more strongly innervated by DA neurons than its first-order counterpart, reaching the levels of highest cortical density in some nuclei (Sánchez-González et al., 2005). Thalamic DA fibers are much denser in primates than in rodents (García-Cabezas et al., 2009). Dopaminergic afferents in the thalamus stem from multiple sources including the hypothalamus, ventral mesencephalon, the PAG, and the lateral parabrachial nucleus, possibly hinting at the existence of a distinct “thalamic dopaminergic system” (Groenewegen, 1988; Sánchez-González et al., 2005). Interestingly, the densest dopaminergic projections to the thalamus are found in those second-order nuclei that are linked to frontal and limbic cortex, e.g., the mediodorsal (MD) nucleus and the midline nuclei (García-Cabezas et al., 2007). In fact, these DA projections share many anatomical features with the strong meso-prefrontal DA system (Melchitzky et al., 2006). These findings suggest that DA could modulate higher-order frontal lobe functions both at the cortical level and by controlling its associated thalamic nuclei (Varela, 2014). Finally, it has been noted that VTA axon terminals and terminals from thalamic MD neurons converge in mPFC layer 5, forming a synaptic triad as described above (Kuroda et al., 1996). Thus, dopaminergic modulation of thalamic function might also have extra-thalamic components.

The main auditory midbrain nucleus, the IC, is targeted by TH-immunoreactive nerve terminals arising from the subparafascicular thalamic nucleus (Tong et al., 2005; Nevue et al., 2015), and the IC has been shown to express D2 receptors (Weiner et al., 1991). Dopaminergic afferents to the superior colliculus, a crucial midbrain structure for oculomotor and visual processing, are sparser in comparison (Weiner et al., 1991).

### Dopaminergic Modulation of Sensory Physiology

Dopamine neuron activity is classically described as being time locked to the presentation of rewarding stimuli. Phasic firing of action potentials by DA neurons especially in the medial mesencephalon communicates a reward prediction error that scales with the difference between predicted and actual reward (Schultz, 2007). These neurophysiological findings form the basis for the large number of studies that have investigated DA’s role in motivation, appetence, and reward-related learning (“motivational salience”). More recent experiments have revealed the existence of functionally distinct subgroups of DA neurons in particular in the lateral midbrain that are activated both by rewarding and aversive events (Matsumoto and Hikosaka, 2009; Matsumoto et al., 2013). This suggests that these neurons are responsive to a more general group of sensory stimuli that are behaviorally relevant and should trigger an appropriate coordinated response (“cognitive salience”). The physiological effects of DA on sensory information processing, however, are not yet well understood. Given the sparse innervation of sensory structures, comparatively few studies have addressed the role of DA in directly modulating sensory inputs (**Table 6**).

**Table 6:** Findings related to sensory modulation by DA.

Structure	Species and anesthesia (if applicable)	Significance	Reference
V1	Remifentanil anesthetized rhesus monkey	Systemic administration of L-DOPA increases supragranular oscillatory activity encoding visual information, but failure to induce changes in neuronal activity by local DA application	Zaldivar et al., 2018, 2014
dLGN	Urethane anesthetized rat and ketamine or halothane anesthetized cat	D1 receptors inhibit and D2 receptors excite extracellularly recorded relay neurons	Phillis et al., 1967; Albrecht et al., 1996; Zhao et al., 2002
dLGN	Rat and mouse (brain slices)	D1 receptors depolarize and D2 receptors inhibit intracellularly recorded neurons	Govindaiah and Cox, 2005; Munsch et al., 2005
A1	Awake gerbil	D1 receptors modulate auditory discrimination learning	Schicknick et al., 2012; Happel et al., 2014
MGN	Mouse (brain slices)	D2 receptors modulate synaptic transmission at thalamocortical afferents in A1	Chun et al., 2014
Inferior colliculus	Awake mouse	Dopamine inhibits neuronal activity	Gittelman et al., 2013
S1	Awake rat	Dopamine inhibits neuronal activity	Bassant et al., 1990
Ventrobasal thalamus	Rat (brain slices)	Dopamine increases excitability of intracellularly recorded neurons	Govindaiah et al., 2010a


In rat and cat dorsal LGN, *in vivo* iontophoretic activation of D1 receptors produced inhibition, whereas engagement of D2 receptors resulted in excitation of extracellularly recorded relay neurons (Phillis et al., 1967; Albrecht et al., 1996; Zhao et al., 2002). Intracellular recordings in rat and mouse LGN slices, however, found that D1 receptors lead to an excitatory membrane depolarization in relay neurons (Govindaiah and Cox, 2005), whereas D2 receptors on local GABAergic interneurons produced inhibition in postsynaptic neurons (Munsch et al., 2005), in complete contrast to the *in vivo* results. It is currently unclear whether these diverging findings reflect dose-dependent effects, where application of small and large drug concentrations often yields opposite effects (Zhao et al., 2002), or rather result from network mechanisms that differ in the *in vivo* and *in vitro* preparation.

In primate primary visual cortex (V1), oscillatory activity (local field potentials) containing information about animated visual stimuli (movie clips) was enhanced in particular in supragranular layers following systemic administration of the DA precursor L-DOPA (Zaldivar et al., 2018). However, focal application of DA failed to induce changes in neuronal activity (Zaldivar et al., 2014), possibly reflecting the very sparse dopaminergic innervation of the occipital pole. In accordance with the notion that dopaminergic effects on visual processing are not mediated by early primary visual regions but possibly by higher-order brain areas, visually induced neuronal responses were directly modulated by DA in the monkey lateral PFC (Jacob et al., 2013). Here, iontophoretically applied DA affected two distinct neuronal populations involved in encoding behaviorally relevant visual stimuli. In putative interneurons, DA inhibited activity in form of a subtractive shift in response levels with unchanged SNR. In putative pyramidal neurons, DA increased excitability in form of a multiplication in gain and enhanced SNR through a reduction of response variability across trials (Jacob et al., 2013). By increasing the coding strength of sensory signals, DA could play a crucial role in how sensory information is represented, memorized, and interpreted in PFC (Ott et al., 2014; Jacob et al., 2016).

Acting on cognitive brain centers, DA might influence visual cortical processing (Arsenault et al., 2013) by long-range interactions originating, e.g., in frontal cortex. Support for this hypothesis comes from exploring DA’s modulatory influence on PFC-guided allocation of visual attention in the macaque monkey (Noudoost and Moore, 2011). D1R were blocked by local antagonist injections into sites of the frontal eye fields (FEFs) that represented the same region of visual space (the “response field”) as simultaneously recorded neurons in visual cortex area V4. Prefrontal D1R antagonism caused the animals to saccade more frequently toward FEF response field targets, meaning this part of the visual field had grasped their attention. The response properties of V4 neurons were changed in a way that is consistent with a multiplicative increase in gain: first, there was an enhancement in the magnitude of responses to visual stimulation; second, the visual responses became more selective to stimulus orientation; third, the visual responses became less variable across trials (Noudoost and Moore, 2011). Thus, prefrontal top-down control over visual cortical neurons during visual attention is under the influence of D1 receptors.

In the auditory system, both inhibitory and – less frequently – excitatory responses in IC neurons were observed after local iontophoretic application of DA in awake mice (Gittelman et al., 2013). The importance of subcortical DA for auditory information processing was underscored by showing that overexpressed MGN D2 receptors in a schizophrenia mouse model reduce the efficiency of synaptic transmission of excitatory thalamocortical afferents in auditory cortex (Chun et al., 2014). These changes were accompanied by behavioral deficits in auditory perception (acoustic startle response). Because thalamocortical afferents in visual or somatosensory cortex were not affected, studying this mouse model might help to provide a neuronal mechanism that links dopaminergic dysfunction to the generation of auditory hallucinations in schizophrenia (Chun et al., 2014). Auditory processing is modulated by cortical DA receptors as well. Systemic administration of D1 receptor antagonists or local injections directly into auditory cortex of gerbils impaired discrimination learning of acoustic stimuli, while D1 receptor agonists improved the animals’ performance (Schicknick et al., 2012; Happel et al., 2014).

Finally, the representation of somatosensory information is also strengthened by DA: *in vivo* depletion of DA in mouse striatum worsened the laterality coding of whisker deflections in medium spiny neurons, i.e., the projection neurons of this structure, indicating that tactile acuity requires dopaminergic input (Ketzef et al., 2017). Intracellular recordings in rat VB thalamic slices showed that DA increases neuronal excitability in two different ways: activation of D1 receptors leads to membrane depolarization, whereas D2 receptors facilitate action potential discharge (Govindaiah et al., 2010b). In contrast, in rat S1, iontophoretic DA application produced inhibitory effects on neuronal firing (Bassant et al., 1990).

### Putting It All Together: Monoaminergic Neuromodulation of Sensory Processing

The discussed studies provide compelling evidence that the representation and processing of sensory information is heavily regulated by the brain’s monoamine transmitters. Despite their widespread cortical and subcortical targeting, the monoaminergic systems are by no means blurry, brain-wide modulators of neuronal activity. They send regionally and laminar-specific projections, which may allow them to control both feed-forward and feed-back influences (Cumming and Nienborg, 2016) on sensory signaling by distinct mechanisms. These regional and laminar differences are particularly pronounced in the macaque brain (**Figure 1**).

The indolamine serotonin has prominent projections to the primary sensory areas and innervates all cortical layers including the thalamo-cortical input layer 4. Serotonin is well suited to regulate the sensory input stages by acting in these primary sensory areas and in the first-order thalamic relay nuclei. Despite some degree of variability between studies on the serotonergic modulation of sensory processing, the emerging pattern is that it dampens neuronal responses and reduces gain. The reduction in gain may reduce the salience of a sensory stimulus (Seillier et al., 2017). Alternatively, if it only affects the spontaneous but not the stimulus-driven response (Lottem et al., 2016), it may increase a neuron’s SNR and hence effectively increase stimulus salience. Probing the serotonergic modulation of sensory processing during perceptually driven behavior may resolve this seeming discrepancy. In general, however, the experimental data show that serotonin directly modulates sensory processing as early as the feed-forward sensory input stage.

In contrast to serotonin the catecholamine DA has a pronounced fronto-occipital gradient (**Figure 1**), is less abundant in sensory cortices, particularly sparse in the primary visual cortex, and only weakly active in granular layers. This suggests that dopaminergic effects on sensory processing are not mediated primarily by local modulation of early sensory input stages (e.g., Zaldivar et al., 2014) but instead by modulating long-range recurrent cortico-cortical and cortico-bulbar interactions originating in the strongly innervated supragranular and infragranular layers, respectively, e.g., of the frontal lobe cognitive control centers (Lewis et al., 1987; Noudoost and Moore, 2011; Jacob et al., 2013). Its modulatory effects stem from a variable and complex combination of inhibition and excitation in the targeted circuits. The net result is likely an increase in SNR, which is based both on additive operations, e.g., predominant suppression of responses to non-preferred stimuli (“sculpting inhibition”) (Vijayraghavan et al., 2007), and on multiplicative operations, e.g., increase in gain and response reliability (reduction of trial-to-trial variability) (Noudoost and Moore, 2011; Jacob et al., 2013). Thus, the role of DA could primarily be to adapt the read-out of sensory circuits to best serve task demands and behavioral goals.

The catecholamine NA, conversely, may play a role in between these two extremes. It has a less pronounced fronto-occipital gradient than DA and, like serotonin, substantial projections to the primary sensory cortices, including the primary visual cortex. But in contrast to serotonin, the innervation of the granular layers is relatively sparse (**Figure 1**). The modulatory effects of NA on sensory processing are diverse. This variability may reflect adaptable modulation depending on the behavioral state of the animal, in line with the notion of optimizing the neuronal gain for behavior (Aston-Jones and Cohen, 2005).

Taken together, the current literature argues for a prominent complementarity in sensory neuromodulation by monoamines. This complementarity is prominent anatomically (**Figure 1B**), resulting in both direct bottom-up and indirect top-down control over sensory signaling by the indolamine serotonin and the catecholamines NA and DA, respectively (Papadopoulos and Parnavelas, 1991). One of the main challenges will now be to dissect the individual contributions of the anatomically and functionally separable monoamine subsystems in shaping how sensory information is represented, processed, and evaluated by the brain’s sensory, cognitive, and motivational networks (**Figure 1A**). We believe that key to such insights will be the combination of increased specificity and precision when targeting these neuromodulatory systems with well characterized behavior (Krakauer et al., 2017).

## Author Contributions

All authors listed have made a substantial, direct and intellectual contribution to the work, and approved it for publication.

## Conflict of Interest Statement

The authors declare that the research was conducted in the absence of any commercial or financial relationships that could be construed as a potential conflict of interest.
